# *Besnoitia besnoiti* among cattle in insular and northwestern Italy: endemic infection or isolated outbreaks?

**DOI:** 10.1186/s13071-014-0585-4

**Published:** 2014-12-10

**Authors:** Alessia L Gazzonis, Gema Alvarez Garcia, Sergio A Zanzani, Giovanni Garippa, Luca Rossi, Marco Maggiora, Valter Dini, Anna Invernizzi, Mario Luini, Vito M Tranquillo, Luis Ortega Mora, Maria Teresa Manfredi

**Affiliations:** Department of Veterinary Science and Public Health, University of Milan, Milan, Italy; SALUVET, Animal Health Department, Faculty of Veterinary Sciences, Complutense University of Madrid, Madrid, Spain; Department of Veterinary Medicine, University of Sassari, Sassari, Italy; Department of Veterinary Sciences, University of Turin, Turin, Italy; Health Veterinary Service, A.S.L. Savonese 2, Savona, Italy; Istituto Zooprofilattico Sperimentale della Lombardia e dell’Emilia Romagna, Milan, Italy; Istituto Zooprofilattico Sperimentale della Lombardia e dell’Emilia Romagna, Lodi, Italy; Istituto Zooprofilattico Sperimentale della Lombardia e dell’Emilia Romagna, Bergamo, Italy

**Keywords:** *Besnoitia besnoiti*, Apicomplexa, Protozoa, Bovine besnoitiosis, ELISA, Western blot, Beef cattle, Dairy cattle, Italy

## Abstract

**Background:**

Bovine besnoitiosis, caused by the apicomplexan *Besnoitia besnoiti*, is a chronic and debilitating disease considered as emerging in Europe. In Spain, Portugal and France it is endemic and foci of infection were recorded in Germany, Switzerland, Hungary, Greece and Italy. In Italy, cases of bovine besnoitiosis were registered both in imported and autochthonous cattle, and mostly in central regions; high seroprevalence was also revealed by an epidemiological survey performed in the southern part of the country. Aiming to update information on the disease in northwestern and insular areas of Italy, where data on bovine besnoitiosis were missing, a serosurvey was designed for the present study.

**Methods:**

Three thousand one hundred and forty bovine blood samples from both dairy and beef farms (*n* = 126) were collected in northwestern regions (Lombardy, Piedmont and Liguria) and in the island of Sardinia. Samples were analyzed by a standardized in-house ELISA and those resulted positive were re-tested by Western Blot (WB) for confirmation. On results obtained by both ELISA and WB, apparent (AP) and true prevalence (TP) were calculated at individual and herd levels. Further, a panel of sera resulted positive to ELISA was analyzed by IFAT.

**Results:**

A total of 712 animals (AP = 22.7%; TP = 18.8%) and 109 farms (AP = 86.5%; TP = 88.2%) showed a positive reaction in ELISA. Only ten (AP = 0.3%; TP = 0%) specimens proceeding from five farms (AP = 3.9%; TP = 1.7%) from Lombardy were confirmed positive to the WB, corresponding to two Holstein Friesian cows and eight beef cattle. IFAT showed a low sensitivity (44.4%) scoring positive in only four samples out of 9 positive to WB.

**Conclusions:**

The survey demonstrated that bovine besnoitiosis cannot still be considered endemic in whole Italy. In fact, independent foci of infection were registered only in Lombardy region. Therefore, a sanitary strategy aimed to increase control measures and to organize monitoring plans, by adequate diagnostic tools is necessary to avoid overestimation of *B. besnoiti* in Italy.

## Background

*Besnoitia besnoiti* is a protozoan parasite belonging to the group of cyst-forming coccidians (Apicomplexa, Sarcocystidae) related to *Toxoplasma gondii* and *Neospora caninum*. Similarly to other *Besnoitia* species infecting ungulates, the life cycle of *B. besnoiti* is in part unknown: cattle represent the intermediate host, whereas the definitive host, if any, has not yet been identified. By analogy with other apicomplexan protozoa, a carnivore, possibly the cat, has been suggested as the definitive host [1,2]. Hematophagous insects (*Glossina*, *Stomoxys* and Tabanids) are considered potential mechanical vectors [3]. Moreover, a close contact between animals or an incorrect medical procedure (e.g. a repeated use of hypodermic needles) have been suggested as potential means of transmission of the infection [1,4]. Animal trade and movement throughout countries have been identified as major risk factors for establishment of new bovine besnoitiosis (BB) foci in naive areas and countries [4]. Furthermore, the role of wild animals as possible hosts of the parasite needs to be investigated; hitherto, only few cases of seropositivity in red deer and roe deer have been registered in Europe and any surveyed wild carnivores showed antibodies against *B. besnoiti* [5,6]. In Europe, BB is considered an emerging or re-emerging disease, with increasing geographical distribution and caseload. It is endemic in large areas in Spain, Portugal and France, while isolated outbreaks have been reported in Germany, Switzerland, Italy, Greece and Hungary [4,7-13]. In Italy, besides cases in imported cattle [14,15], autochthonous outbreaks involving local breeds and/or native individuals of any breed have been reported in the central mainland part of the country [15-19]. In contrast with the focal distribution of these outbreaks, two ELISA-based surveys revealed high seroprevalence values in southern Italy (44.1% and 83% at individual and farm level respectively) [20] and central Italy (29.4-52% and 94.6-100% at individual and farm level, respectively) [21]. Earlier, Gentile *et al*. [16] considering the recurrence of a few besnoitiosis outbreaks and the high seroprevalence values in an infected farm hypothesized that besnoitiosis should be retained endemic in Italy.

According to EFSA [7], epidemiological surveys are recommended to monitor the spread of *B. besnoiti* in Europe and to increase knowledge on its biology and associated risk factors. Several standardized diagnostic techniques have been developed such as ELISA, IFAT, MAT and Western Blot and a few of them were recently validated in a European inter-laboratories trial. Particularly, in order to increase test performance and to obtain valuable epidemiological data, the combination of ELISA with a posteriori more specific technique have been recommended [4,22]. We designed a cross-sectional survey to investigate the seroprevalence of *B. besnoiti* in areas of Italy not much yet examined: northwestern Italy and Sardinia Island, representing a huge variety of geographical and ecological features. Our main goal was contributing to a reliable representation of *B. besnoiti* distribution at national scale, and to the debate on tools for active surveillance of BB in Europe.

## Methods

### Area description

The serosurvey was carried out in two separate areas: mainland northwestern Italy (including Lombardy, Piedmont and Liguria regions) and insular Italy (Sardinia Island). Sampled areas were representative of a high variety of management systems and of differences in landscape and climate.

Northern regions in Italy host mainly intensive farms for calf and beef production (an average of 800 animals per farm); the majority of farms from Lombardy and Piedmont are mainly located in the flatland of River Po Valley, whose continental climate is characterized by very cold winter and hot-moist summer. The overall cattle population is of 1,484,000 and 815,000 in Lombardy and Piedmont, respectively, and the purchase of spare breeding animals is mainly from abroad (292,593 in Lombardy and 226,147 in Piedmont representing 22.1% and 17.1%, respectively, of imported cattle in Italy in 2010), particularly from France.

Liguria is a narrow region bordered by the mountains (the Alps and the Apennines) and the Ligurian Sea; thanks to these geographical features, its climate is quite mild all year round. In this region, farms are smaller (an average of 20 animals per farm) and located mostly in the central western area; beef breeding is more represented than dairy for an overall of 14175 cattle and only 112 animals were imported from abroad. Sardinia is an island with an area of 20,000 km^2^ located West to the Italian peninsula in the Mediterranean Sea whose farming activity is characterized by few exchanges of animals with the continental regions. In 2010, only 385 cattle were imported there from foreign countries. The number of bred cattle is very low, amounting to 251,000 heads. Data were obtained from ISTAT [23].

### Study population and sample collection

A cross-sectional study was carried out using the individual animal as the sampling unit. Farms in the study were stratified by productive category (dairy and beef) and then randomly selected from those included in the National Plan for the control of bovine brucellosis. Sampling stratification was performed on the basis of administrative boundaries; therefore, a minimum sample size for each sampled region was determined by Winepiscope 2.0 (http://www.winepi.net/uk/index.htm) to exclude (in case of all samples negative) a *B. besnoiti* seroprevalence ≤50% within the animals in the sampled herds at a confidence level of 95% and an error margin of 5%. Data on animal amounts were obtained by ISTAT [23].

Within each selected herd, animals over 12 months were sampled by systematic random selection, proportionally to the number of animals present in the farm (mean 25; min-max: 15–75). Both dairy and beef farms were selected and included in the survey. On the whole, 3140 bovine blood samples from 126 farms (79 from Lombardy, 12 from Piedmont, 15 from Liguria and 20 from Sardinia) were collected between October 2012 and May 2013 by local veterinarians in conjunction with routine sampling for regional sanitary controls. Different breeds were sampled: Holstein Friesian and Piedmontese breeds were the most consistent, followed by other cosmopolitan (i.e. Charolaise and Limousine) and local breeds (i.e. Italian Brown, Bruno-Sarda and Grey Alpine). GPS (Global Positioning System) coordinates of each farm were gathered to map its location. No signs of besnoitiosis or other clinical signs were signaled by veterinarians in sampled hosts. At sampling time, individual data on each sampled animal (gender, age, and breed) and on farm management (dairy or beef farm) together with the origin of animals (born in farm, bought from another Italian farm or abroad) were recorded.

Blood samples were collected from jugular or tail vein, kept in tubes without anticoagulant agents and transported to the laboratory in few hours, then centrifuged (15 min, 2120 g). Sera were stored at −20°C until analyzed.

### Serology

Serum samples were analyzed for antibodies against *B. besnoiti* by an in-house ELISA standardized at the Animal Health Department (SALUVET) of the “Complutense” University of Madrid [24]. To confirm the results, the sera tested positive in ELISA were later analyzed by Western Blot (WB). The ELISA and WB used in the present survey showed a sensitivity of 97.3% and 98.1% and a specificity of 94.6% and 97.7%, respectively [22]. As control for both tests, positive and negative sera samples previously tested by IFAT and WB were used [24]. Further, a panel of sera resulted positive to ELISA was analyzed by IFAT [25].

### ELISA

Sera were analyzed through a standardized in-house ELISA as previously described [24]. Samples were analyzed in duplicate, and the mean value of the optical density (OD) was converted into a relative index per cent (RIPC) by employing the following formula:

RIPC = (OD sample - OD negative control)/(OD positive control - OD negative control) × 100. Samples with an RIPC ≥ 9.7 were considered positive.

### Western blot

Western Blot (WB) was performed under non-reducing conditions as previously described [25,26]. Images from the membranes were obtained using a GS-800 Scanner (Bio-Rad Laboratories, CA, USA) and analyzed with Quantity One1 quantification software v. 4.0 (Bio-Rad Laboratories, CA, USA). Samples were considered positive if presented minimum three bands in at least two of the following areas: area I (72.5, 58.9 and 51.4 kDa), area II (38.7, 31.8 and 28.5 kDa) and area III (23.6, 19.1, 17.4 and 14.5 kDa).

### Immunofluorescence assay (IFAT)

A panel of 61 sera, including nine sera confirmed positive to WB, resulted positive to ELISA was processed by IFAT with cut-off titer of 1:200 as described by Fernandez-Garcia *et al*. [25].

### Data analysis

Apparent (AP) and true prevalence (TP) were calculated based on ELISA and WB results, both at individual and herd level [27]. A farm was considered positive if at least one seropositive animal was found. A multivariate binary logistic regression analysis was performed on WB results to determine factors that could be considered predictors of seropositivity. Both individual and farm data were included in the analysis as independent variables: breed, origin (born in farm, born in another Italian farm, imported from abroad), age, region, production (dairy or beef). Gender was not included because of the numerical disproportion between males and females. Statistical analysis was performed by SPSS (version 21.0; SPSS, Chicago, IL). Sensitivity (SE), Specificity (SP), Positive predictive (PPV) and Negative predictive values (NPV) for IFAT were calculated using WB results as gold standard. Further, the agreement between the two serological assays was determined by Youden’s test (Winepiscope 2.0, http://www.clive.ed.ac.uk/winepiscope/0).

### Ethical statement

The survey has been approved by the Ethics Committees of all involved institutes; the sampling was performed respecting the Italian animal welfare regulations.

## Results

**Figure 1:**
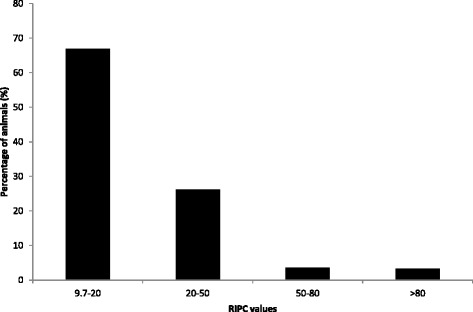
**RICP values distribution in 712 seropositive cattle to** ***Besnoitia besnoiti*** **by ELISA.**

In ELISA, anti-*Besnoitia* antibodies were revealed in 712 out of 3140 samples (AP = 22.7%; TP = 18.8%). The RIPC values showed a high variability with most of the samples (66.9%) presenting low values comprised between 9.7% (i.e. cut-off value) and 20%, 26.3% moderate values, 3.7% moderate-high values whereas very few animals (3.4%) had higher RIPC values (>80) (Figure 1). Of the positive cattle, 127 were beef cattle (AP = 10.6%; TP = 5.6) and 585 were dairy cattle (AP = 30.1%; TP = 26.9%). Overall, 109 farms (AP = 86.5%; TP = 88.2%) housed at least one ELISA seroreactor (Table 1). In particular, two dairy breeds, Italian Brown and Holstein Friesian, presented higher seroprevalence (27.3% and 38.1%, respectively) in comparison to the other breeds, whose prevalence values ranged from 9.2% to 12.2%. Considering the geographic regions, Lombardy presented the highest number of seropositive animals (27.9%) differently from Piedmont (10.4%), Liguria (13%) and Sardinia (12.2%).

**Table 1:** **Diagnostic of** ***Besnoitia besnoiti*** **infection in cattle from northwestern and insular Italy by serological analysis (ELISA and WB)**

	**Production category (N° samples)**	**Serology test**	**N° positive**	**AP**	**95% CI**	**TP**	**95% CI**
Individual level	Overall (3140)	ELISA	712	22.7	21.2-24.1	18.8	17.4-20.1
		WB	10	0.3	0-0.7	0	0-0
	Dairy (1941)	ELISA	585	30.1	28.1-32.2	26.9	25-28.8
		WB	2	0.1	0-0.2	0	0-0
	Beef (1199)	ELISA	127	10.6	8.8-12.3	5.6	4.-7.2
		WB	8	0.7	0.2-1.1	0	0-0
Farm level	Overall (126)	ELISA	109	86.5	80.5-92.5	88.2	82.7-93.7
		WB	5	3.9	0.6-7.4	1.7	0-5
	Dairy (77)	ELISA	77	100	95.3-100	100	95.3-100
		WB	2	2.6	0-6.1	0.3	0-3.7
	Beef (49)	ELISA	34	69.4	56.5-82.3	69.6	57.7-81.5
		WB	3	6.1	0-12.8	4	0-10.4

**Figure 2:**
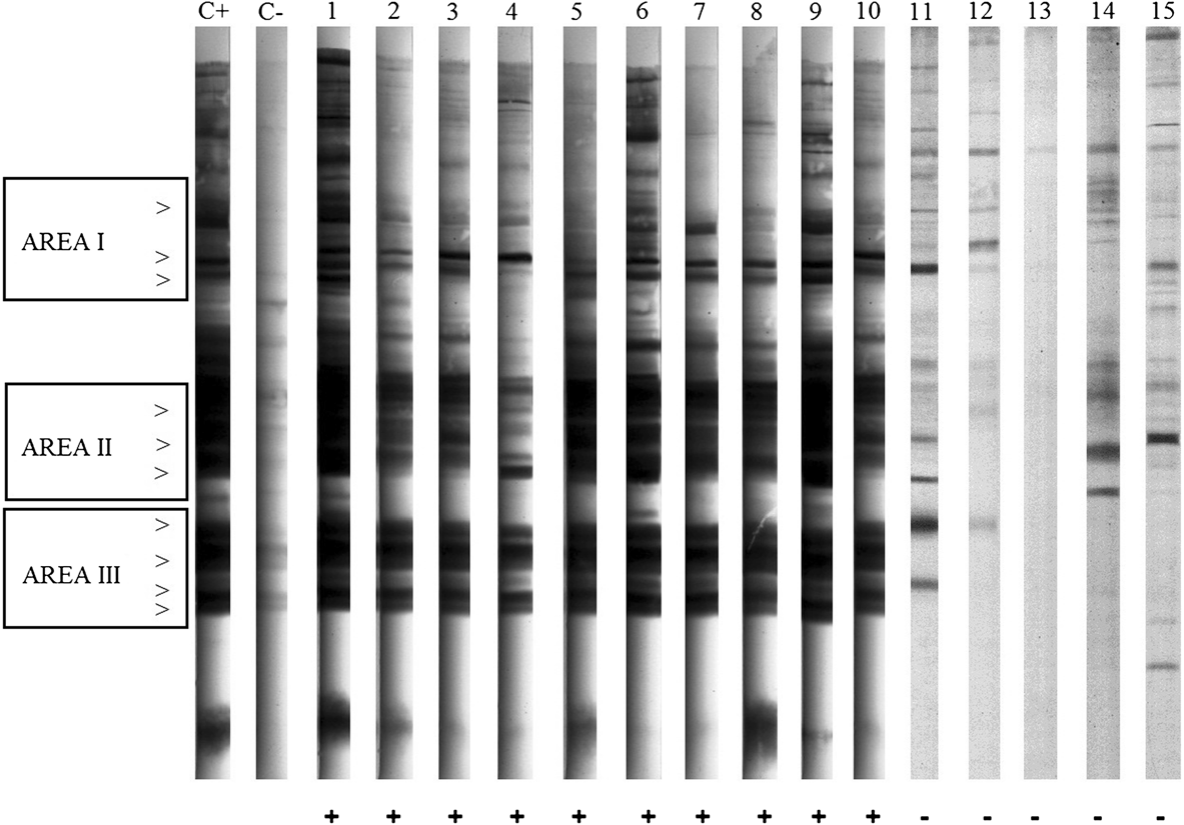
**Pattern of recognition of** ***Besnoitia besnoiti*** **tachyzoite antigens by sera from naturally infected cattle by Western Blot.** Antigenic bands in the three main antigenic areas are indicated by arrows. C+: positive control; C-: negative control. Lanes 1–10: positive samples (as indicate in Table 2). Lines 11–15: negative samples.

**Figure 3:**
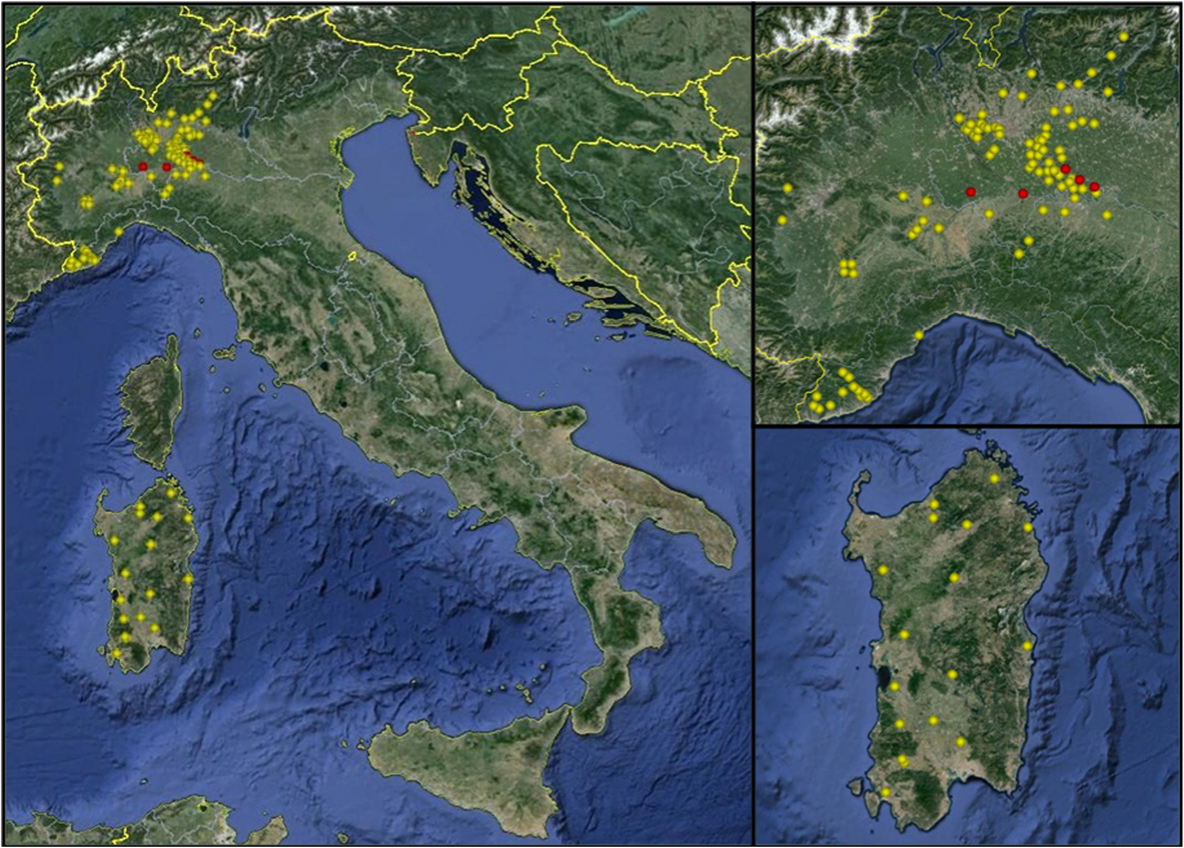
**Distribution of farms of cattle sera tested for specific antibodies to** ***Besnoitia besnoiti*** **in the two samples areas: Mainland northwestern Italy (including Lombardy, Piedmont and Liguria regions) and insular Italy (Sardinia Island).** Positive farms are in red.

The WB confirmed the presence of anti-*Besnoitia* antibodies in a minority of ELISA seroreactors; only ten cattle and five farms with AP of 0.3% and 3.9% at individual and farm level, respectively, were found positive with WB. The TP was 0% at individual level and 1.7% at farm level. Samples confirmed positive by WB presented ELISA RIPC values ranging from 50.2 to 202.8 (Table 2). Particularly, out of WB positive animals, five showed ELISA RIPC values of 50–80 and five >80. Cattle serum recognized all immunodominant antigens described in each antigenic area (Figure 2). Risk factors analysis on data obtained by WB produced a non-fitting model as none of the considered independent variables resulted in significant values (P > 0.05). Data on animals and farms testing positive in both ELISA and WB are summarized in Table 2, while the location of all sampled farms are represented in Figure 3. All positive farms were located in Lombardy. Of the ten positive cattle, three were imported from France, five were born in the same farm where sampling was carried out and two were born on other farms in Italy. Five of the seroreactors belonged to the same farm A, a beef cow/calf operation, housing about 700 Limousine adult cattle. Weaning and sale of calves occurs at 6–7 months. Natural mating is practiced and service bulls are mostly imported from France. Farm B is a lairage where cattle of different origin (Italy and a range of European countries) are rested on the way to domestic market. Farms C and D are located quite near the Apennines and farm C is close to a beef cattle farm. They are dairy farms with intensive system housing approximately 400 and 200 Holstein Friesians, respectively; in both farms, artificial insemination (A.I.) is regularly practiced. Finally, farm E is another beef cow/calf operation housing about 100 crossbreds. Cattle from farms A, B and E live in paddocks, whereas cattle from C and D are housed in cubicles. Hygienic sanitary condition and animal welfare are very high in all of these farms.

**Table 2:** **Data on seropositive cattle and corresponding ELISA and Western Blot findings**

**Farm**	**Region**	**Geographic coordinates**	**Altitude (m)**	**Cattle n°**	**Breed**	**Gender**	**Age (months)**	**Production**	Origin*	**Time in the farm (months)**	**ELISA (RICP)** ^**§**^	**WB**
A	Lombardy	45°8’51”36 N; 09°51’20”16 E	41	1	Limousine	Female	159	beef	I	135	130.8	+
				2	Limousine	Female	157	beef	I	137	98.7	+
				3	Limousine	Female	157	beef	I	137	191.9	+
				4	Limousine	Female	95	beef	BF	95	80.6	+
				5	Limousine	Female	126	beef	BI	118	55.5	+
B	Lombardy	45° 6’22.93”N; 9°17’1.10”E	62	6	crossbreed	Female	20	beef	BF	20	73.2	+
				7	Limousine	Female	17	beef	BI	16	68.5	+
C	Lombardy	45°14’55.77”N; 9°37’22.10”E	64	8	Holstein Friesian	Female	38	dairy	BF	38	50.2	+
D	Lombardy	45°11’15.10”N; 9°44’8.93”E	59	9	Holstein Friesian	Female	98	dairy	BF	98	60.4	+
E	Lombardy	45°6’51.96”N; 8°51’54.82”E	89	10	Crossbreed	Female	14	beef	BF	14	202.8	+

*I = Imported; BF = Born in the farm; BI = Born in another Italian farm.

§ = cut-off > 9.7.

In the IFAT, only eight sera (8/61, 13.1%) resulted positive at the threshold value of a dilution of 1:200. IFAT scored positive in only four samples out of 9 positive to WB (Table 3). The IFAT performances were evaluated using WB results as gold standard; the test showed SE = 44.4% (95% CI: 11.9-76.9%), SP = 92.3% (95% CI: 85.1-99.5%), PPV = 50% (95% CI: 15.3-84.6%) and NPV = 90.5% (95% CI: 82.7-98.4%). The agreement between IFAT and WB resulted low (0.367).

**Table 3:** **Comparison between serological analysis by IFAT and WB findings (gold standard) on a panel of 61 sera resulted positives in ELISA**

	**WB anti-** ***B. besnoiti***		
**IFAT anti-** ***B. besnoiti***	**Positive**	**Negative**	**Total**
Positive	4	4	8
Negative	5	48	53
Total	9	52	61 (n)

## Discussion

In recent years, EFSA recognized bovine besnoitiosis as an emerging or re-emerging infection in Europe [7]. Endemic BB areas have been signaled in Spain, Portugal and France, however only isolated cases/outbreaks have been reported in central and eastern European countries; in a few cases the infected animals were imported from France [8,28,29]. In Italy, the first cases of BB were diagnosed two decades ago in imported beef cattle [15], but reports of it in autochthonous breeds date back to recent years [17-19].

The present serosurvey was designed to investigate the diffusion of *B. besnoiti* among beef and dairy cattle in regions in mainland and insular Italy (Sardinia) poorly scanned. Sampled farms were deemed representative of different geographical, ecological and management scenarios. Considering our data, BB seemed to be limited to sporadic and independent foci of infection in Lombardy region. In other regions belonging to northwestern Italy, Piedmont and Liguria, any of sampled sera showed reaction to *B. besnoiti* in WB. Cases of BB were previously registered in Piedmont [14,15], therefore we cannot exclude the presence of foci of infection in areas or herds not included in the survey. Of particular interest is the absence of BB in Sardinia, probably thanks to its geographical features and to the limited exchange in the purchase of spare breeding animals that contributes to prevent the spread of infections from the continental areas.

In our study, the high prevalence and wide geographical distribution of seroreactors in ELISA (22.7%) clearly conflicts with the limited number of WB positive samples (0.3%). The low BB prevalence is also not consistent with the results of previous ELISA-based serosurveys carried out in central and southern Italy [16,20]. In an inter-laboratory comparative study, high sensitivity and specificity were registered for many commercial and in-house ELISA tests [22]. However, other authors documented a high rate of false positives in ELISA, and recommended the complementary use of robust confirmation tests on occasion of BB serosurveys [4,29,30]. Similarly to our study, 10% of investigated cattle in Switzerland tested positive with a commercial ELISA but only 0.3% was later confirmed WB positive [29]. In Australia, the same commercial ELISA yielded 18% seropositive cattle but was not confirmed in WB, and the Authors concluded that *B. besnoiti* was absent in the country [31]. False-positive results may be due to cross-reactions with closely related Apicomplexa such as *Sarcocystis* spp., *Toxoplasma gondii* and *Neospora caninum* that are known to potentially cross-react with *Besnoitia* spp. [25,30,32-34]. Furthermore, in the present study most ELISA positive sera had RIPC values comprised between 9.7 and 20, suggesting low antibody titres in the majority of seroreactors. As opposite, most sera which were analyzed with the same ELISA in a BB endemic area in Spain showed RIPC values comprised between 20 and 80 [10].

Further, we analyzed a panel of sera by IFAT and a comparison with results obtained in WB was performed. IFAT showed a very low sensitivity (44.4%) and PP value (50%) demonstrating that in the surveyed area this serological test would be poorly able to detect all true positive sera. Otherwise, IFAT allowed increasing specificity (92.3%), thus avoiding a major number of false positives in comparison to ELISA. Then, according to a few Authors [29], both IFAT and the ELISA test could be used for screening purposes, with confirmation of positive results by WB. However, ELISA appeared more adequate for large screening whereas IFAT for analysis at individual level. IFAT was confirmed as a more time consuming and a more subjective technique in comparison to ELISA and the choice of coupling a standardized ELISA with a confirmatory WB resulted in a reasonable strategy to carry out epidemiological studies on a large scale in non-endemic areas [4,30].

In spite of the limited geographical distribution of *B. besnoiti* in the investigated areas, its circulation was intense in infected farm A (Table 1), where repeated clinical cases were recorded in a relatively short time interval [35]. In this farm, the 22% prevalence of ELISA seroreactors, later confirmed by WB, compares favorably with similar screenings carried out in outbreak farms in Italy [16] and other non-endemic areas in Europe [4,8]. In similar farms, testing for BB should be mandatory on new entries and on the whole herd in order to control the diffusion of infection in and outside the farm. According to Alvarez-Garcia *et al*. [4], a few measures, such as the employment of seronegative bulls in natural mating and culling of seropositive or with clinical signs animals, should be adopted to an effective control of this infection.

Two out of five confirmed positive farms housed Holstein Fresians. Most BB outbreaks in Europe were recorded in beef farms and the majority of the serosurveys carried out so far focused on beef cattle [8,16-19,28,29]. However, a higher susceptibility of beef cattle to *Besnoitia* infection has not been demonstrated [20,36]. It is reasonable to assume that beef cattle, which are more frequently raised outdoor, are at greater risk of exposure to the bite of putative insect vectors [37].

Transmission through direct contact during natural mating has been also hypothesized [4,38]. In farm A, where natural mating is practiced, bulls in service were imported from BB endemic areas abroad.

Transportation of cattle across areas and countries is a well recognized risk factor for BB [4]. In the present survey, only three positive animals were imported (Farm A), but the origin of the infection could not be inferred since it was impossible to know whether the animals had already been immunized or had acquired the infection once in Italy. Nonetheless, it is reasonable to infer that *Besnoitia* infection must be related to the import of subclinically infected cattle into a farm followed by local transmission. Moreover, farm B was a lairage, a kind of farm where animals from different origins are joined, which increases sanitary risks and makes infections possible. Regarding the other positive animals born in Italy, they might have got infected through close contact with imported animals or by other ways of transmission, such as mechanical vectors. In fact, positive farms are located quite near the Apennines, suggesting that the area could represent an ideal habitat for insect vectors contributing to spread the infection in Italy.

## Conclusions

The survey demonstrated that, while BB remains non-endemic in three out of four investigated regions, foci of infection are present in Lombardy, the main dairy cattle area countrywide. Based on the results, awareness by local practitioners and veterinary officers should be increased to facilitate the early detection of clinical cases and the implementation of sensible control measures (e.g., elimination of infected animals, a ban on free translocations of live cattle originating from outbreak farms) and monitoring plans (i.e. serological control of imported animals). Furthermore, data suggests that surveys in areas with unknown BB prevalence should not be carried out with serological tests defecting in specificity, in order to avoid overestimation or even unsupported conclusions on the presence/absence of *B. besnoiti*.

## Abbreviations

BB: Bovine besnoitiosis
AP: Apparent prevalence
TP: True prevalence
WB: Western blot
ELISA: Enzyme-linked immunosorbent assay
RIPC: Relative index per cent
OD: Optical density
IFAT: Indirect fluorescent antibody test
SE: Sensitivity
SP: Specificity
PPV: Positive predictive value
NPV: Negative predictive value
MAT: Modified agglutination test
CI: Confidence interval
